# Ethyl 2-(7-oxo-3,5-diphenyl-1,4-diaze­pan-2-yl)acetate

**DOI:** 10.1107/S160053681200757X

**Published:** 2012-03-10

**Authors:** G. Jagadeesan, K. Sethusankar, P. Selvakumar, S. Thennarasu, A. B. Mandal

**Affiliations:** a Department of Physics, Dr MGR Educational and Research Institute, Dr MGR University, Chennai 600 095, India; b Department of Physics, RKM Vivekananda College (Autonomous), Chennai 600 004, India; c Organic Chemistry Division, Central Leather Research Institute, Adyar, Chennai 600 020, India

## Abstract

In the title compound, C_21_H_24_N_2_O_3_, the diazepane ring adopts a chair conformation. The central diazepane ring forms dihedral angles 67.80 (7) and 72.29 (5)° with the two benzene rings. The eth­oxy­carbonyl group is disordered over two conformations with site-occupancy factors of 0.643 (5) and 0.357 (5). In the crystal, inversion dimers linked by pairs of N—H⋯O hydrogen bonds generate *R*_2_^2^(8) loops.

## Related literature

For general background to biological activities of diazepane derivatives, see: Hirokawa *et al.* (1998 ▶). For a related structure, see: Ravichandran *et al.* (2009 ▶). For puckering parameters, see: Cremer & Pople (1975 ▶). For graph-set notation, see: Bernstein *et al.* (1995 ▶).
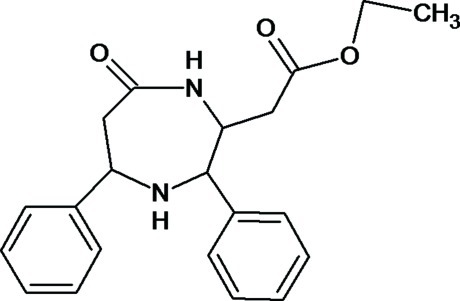


## Experimental

### 

#### Crystal data

C_21_H_24_N_2_O_3_
*M* *_r_* = 352.42
Monoclinic, 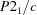

*a* = 10.3721 (19) Å
*b* = 20.666 (4) Å
*c* = 9.1954 (18) Å
β = 104.365 (5)°
*V* = 1909.4 (6) Å^3^
*Z* = 4
Mo *K*α radiation
μ = 0.08 mm^−1^
*T* = 293 K
0.30 × 0.30 × 0.25 mm

#### Data collection

Bruker Kappa APEXII CCD diffractometer
Absorption correction: multi-scan (*SADABS*; Bruker 2008 ▶) *T*_min_ = 0.976, *T*_max_ = 0.980
22045 measured reflections
5000 independent reflections
3284 reflections with *I* > 2σ(*I*)
*R*_int_ = 0.029

#### Refinement

*R*[*F* ^2^ > 2σ(*F* ^2^)] = 0.054
*wR*(*F* ^2^) = 0.163
*S* = 1.03
5000 reflections
259 parameters
H atoms treated by a mixture of independent and constrained refinement
Δρ_max_ = 0.31 e Å^−3^
Δρ_min_ = −0.26 e Å^−3^

### 

Data collection: *APEX2* (Bruker, 2008 ▶); cell refinement: *SAINT* (Bruker, 2008 ▶); data reduction: *SAINT*; program(s) used to solve structure: *SHELXS97* (Sheldrick, 2008 ▶); program(s) used to refine structure: *SHELXL97* (Sheldrick, 2008 ▶); molecular graphics: *ORTEP-3* (Farrugia, 1997 ▶); software used to prepare material for publication: *SHELXL97* and *PLATON* (Spek, 2009 ▶).

## Supplementary Material

Crystal structure: contains datablock(s) global, I. DOI: 10.1107/S160053681200757X/pv2512sup1.cif

Structure factors: contains datablock(s) I. DOI: 10.1107/S160053681200757X/pv2512Isup2.hkl

Supplementary material file. DOI: 10.1107/S160053681200757X/pv2512Isup3.cml

Additional supplementary materials:  crystallographic information; 3D view; checkCIF report

## Figures and Tables

**Table 1:** Hydrogen-bond geometry (Å, °)

*D*—H⋯*A*	*D*—H	H⋯*A*	*D*⋯*A*	*D*—H⋯*A*
N1—H1*A*⋯O1^i^	0.88 (2)	1.97 (2)	2.846 (2)	174 (1)

Symmetry code: (i) 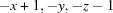.

**Fig. 1.**
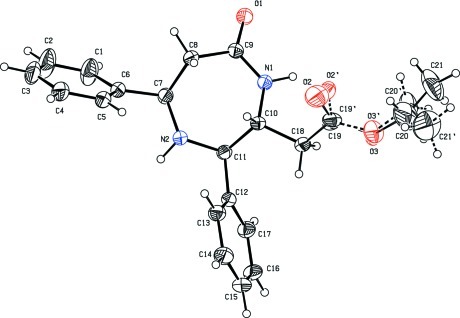
The molecular structure of the title compound with the atom numbering scheme, displacement ellipsoids are drawn at 30% probability level. H–atoms are present as small spheres of arbitary radius. The minor fraction of the disordered ethoxy carbonyl moiety has been represented by broken bonds.

**Fig. 2.**
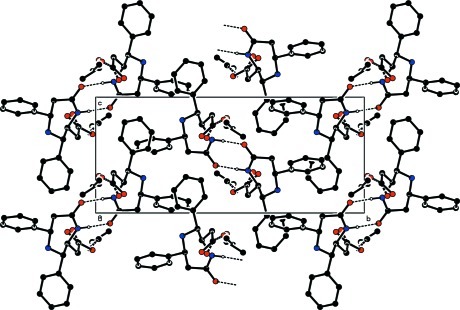
The crystal packing of the title compound viewed down the a axis, showing hydrogen bonds resulting in R22(8) graph-set ring motif. H–atoms not involved in hydrogen bonds have been excluded for clarity.

## supplementary crystallographic information

### Comment

The 1,4-diazepane derivatives are of considerable importance due to their wide spectrum of biological activities (Hirokawa *et al.*, 1998).

In the title molecule (Fig. 1), the central diazepane ring (C7–C11/N1/N2) forms dihedral angles 67.80 (7) and 72.29 (5)° with the two benzene rings (C1–C6) and (C12–C17), respectively; the dihedral angle between the two benzene rings is 55.96 (7)°. The sum of the bond angles around the N1 atom (362.9°) of the diazepane ring is in *sp*^2^ hybridization, whereas the other atom N2 (335.6°) is in *sp*^3^ hybridization.

The diazepane ring adopts a *chair* conformation with puckering parameters (Cremer & Pople, 1975) q_2_ = 0.45 (3) Å, q_3_ = 0.79 (2) Å, φ_2_= 36.8 (2)° and φ_3_= -157.5 (2)°. The title compound exhibits structural similarities with another closely related structure (Ravichandran *et al.*, 2009).

The crystal packing is stabilized by N–H···O intermolecular interaction (Tab. 1 and Fig. 2) which results in a dimer which may be described as an *R*^2^_2_(8) motif in the graphset notation (Bernstein *et al.*, 1995).

The ethoxy carbonyl moiety is disordered with occupancy factors of 0.643 (5):0.357 (5).

### Experimental

Powdered ethyl 2-(4-oxo-2,6-diphenyl piperidin-3-yl)acetate hydrochloride (2 g) was dissolved in an ice cold conc. H_2_SO_4_ placed in a conical flask equipped with magnetic stirrer. After the complete dissolution, the temperature of the solution was brought to 298 K. Sodium azide (600 mg) was added in portions over a period of 20 minutes with vigorous stirring. The solution was then poured slowly on to crushed ice with vigorous stirring, and the pH was adjusted at approximately 8.0 using 4 N sodium hydroxide solution and extracted with chloroform. The combined organic layer was dried over sodium sulfate and evaporated to get the crude product. The crude product was purified by recrystallization from benzene and ethanol (1:1) to afford colourless prismatic crystals of the title compound suitable for X-ray crystallographic studies.

### Refinement

The ethoxy carbonyl moiety was disordered over the positions O2/C19/O3/C20/C21:O2'/C19'/O3'/C20'/C21' with site occupancy factors 0.643 (5):0.357 (5). The hydrogen atoms were placed in calculated positions with C–H = 0.93 to 0.98 Å and refined in the riding model with fixed isotropic displacement parameters: *U*_iso_(H) = 1.5 *U*_eq_(C) for methyl group and *U*_iso_(H) = 1.2 *U*_eq_(C) for other groups. The amino H-atom was allowed to refine freely.

### Crystal data

**Table:**

C_21_H_24_N_2_O_3_	*F*(000) = 752
*M**_r_* = 352.42	*D*_x_ = 1.226 Mg m^−^^3^
Monoclinic, *P*2_1_/*c*	Mo *K*α radiation, λ = 0.71073 Å
Hall symbol: -P 2ybc	Cell parameters from 5000 reflections
*a* = 10.3721 (19) Å	θ = 2.0–28.8°
*b* = 20.666 (4) Å	µ = 0.08 mm^−^^1^
*c* = 9.1954 (18) Å	*T* = 293 K
β = 104.365 (5)°	Block, colourless
*V* = 1909.4 (6) Å^3^	0.30 × 0.30 × 0.25 mm
*Z* = 4	

### Data collection

**Table:**

Bruker Kappa APEXII CCD diffractometer	5000 independent reflections
Radiation source: fine-focus sealed tube	3284 reflections with *I* > 2σ(*I*)
Graphite monochromator	*R*_int_ = 0.029
ω and φ scan	θ_max_ = 28.8°, θ_min_ = 2.0°
Absorption correction: multi-scan (*SADABS*; Bruker 2008)	*h* = −14→12
*T*_min_ = 0.976, *T*_max_ = 0.980	*k* = −27→27
22045 measured reflections	*l* = −12→12

### Refinement

**Table:**

Refinement on *F*^2^	Primary atom site location: structure-invariant direct methods
Least-squares matrix: full	Secondary atom site location: difference Fourier map
*R*[*F*^2^ > 2σ(*F*^2^)] = 0.054	Hydrogen site location: inferred from neighbouring sites
*wR*(*F*^2^) = 0.163	H atoms treated by a mixture of independent and constrained refinement
*S* = 1.03	*w* = 1/[σ^2^(*F*_o_^2^) + (0.0669*P*)^2^ + 0.6968*P*] where *P* = (*F*_o_^2^ + 2*F*_c_^2^)/3
5000 reflections	(Δ/σ)_max_ < 0.001
259 parameters	Δρ_max_ = 0.31 e Å^−^^3^
0 restraints	Δρ_min_ = −0.26 e Å^−^^3^

### Special details

**Table:**

Geometry. All e.s.d.'s (except the e.s.d. in the dihedral angle between two l.s. planes) are estimated using the full covariance matrix. The cell e.s.d.'s are taken into account individually in the estimation of e.s.d.'s in distances, angles and torsion angles; correlations between e.s.d.'s in cell parameters are only used when they are defined by crystal symmetry. An approximate (isotropic) treatment of cell e.s.d.'s is used for estimating e.s.d.'s involving l.s. planes.
Refinement. Refinement of *F*^2^ against ALL reflections. The weighted *R*-factor *wR* and goodness of fit *S* are based on *F*^2^, conventional *R*-factors *R* are based on *F*, with *F* set to zero for negative *F*^2^. The threshold expression of *F*^2^ > σ(*F*^2^) is used only for calculating *R*-factors(gt) *etc*. and is not relevant to the choice of reflections for refinement. *R*-factors based on *F*^2^ are statistically about twice as large as those based on *F*, and *R*- factors based on ALL data will be even larger.

### Fractional atomic coordinates and isotropic or equivalent isotropic displacement parameters (Å^2^)

**Table:**

	*x*	*y*	*z*	*U*_iso_*/*U*_eq_	Occ. (<1)
O2	0.8227 (16)	0.0990 (16)	−0.341 (3)	0.085 (2)	0.643 (5)
O3	0.8931 (6)	0.0099 (2)	−0.1768 (7)	0.0628 (9)	0.643 (5)
C19	0.8114 (11)	0.0628 (7)	−0.2506 (9)	0.0465 (12)	0.643 (5)
C20	1.0024 (8)	−0.0023 (4)	−0.2420 (8)	0.125 (2)	0.643 (5)
H20A	1.0214	0.0374	−0.2889	0.150*	0.643 (5)
H20B	1.0798	−0.0120	−0.1611	0.150*	0.643 (5)
C21	0.9894 (6)	−0.0484 (3)	−0.3406 (8)	0.129 (2)	0.643 (5)
H21A	0.9694	−0.0882	−0.2971	0.193*	0.643 (5)
H21B	1.0709	−0.0531	−0.3713	0.193*	0.643 (5)
H21C	0.9184	−0.0379	−0.4262	0.193*	0.643 (5)
O2'	0.799 (3)	0.092 (3)	−0.348 (6)	0.085 (2)	0.357 (5)
O3'	0.8947 (13)	0.0289 (5)	−0.2026 (14)	0.0628 (9)	0.357 (5)
C19'	0.797 (2)	0.0621 (15)	−0.223 (2)	0.0465 (12)	0.357 (5)
C20'	0.9985 (16)	0.0226 (9)	−0.2859 (16)	0.125 (2)	0.357 (5)
H20C	0.9687	−0.0100	−0.3629	0.150*	0.357 (5)
H20D	1.0023	0.0633	−0.3372	0.150*	0.357 (5)
C21'	1.1038 (13)	0.0098 (6)	−0.2240 (14)	0.129 (2)	0.357 (5)
H21D	1.1322	0.0376	−0.1386	0.193*	0.357 (5)
H21E	1.1618	0.0152	−0.2899	0.193*	0.357 (5)
H21F	1.1067	−0.0344	−0.1911	0.193*	0.357 (5)
C1	0.0824 (2)	0.22542 (12)	−0.4319 (4)	0.0848 (9)	
H1	0.0401	0.1854	−0.4424	0.102*	
C2	0.0089 (3)	0.28135 (16)	−0.4741 (4)	0.1085 (12)	
H2	−0.0825	0.2786	−0.5139	0.130*	
C3	0.0703 (3)	0.34092 (13)	−0.4575 (3)	0.0856 (8)	
H3	0.0205	0.3782	−0.4870	0.103*	
C4	0.2037 (2)	0.34509 (10)	−0.3979 (2)	0.0626 (5)	
H4	0.2452	0.3853	−0.3846	0.075*	
C5	0.27777 (19)	0.28931 (9)	−0.3569 (2)	0.0471 (4)	
H5	0.3691	0.2925	−0.3168	0.056*	
C6	0.21829 (17)	0.22904 (9)	−0.37441 (19)	0.0440 (4)	
C7	0.30111 (16)	0.16807 (8)	−0.33946 (18)	0.0408 (4)	
H7	0.2434	0.1316	−0.3299	0.049*	
C8	0.36452 (19)	0.15567 (9)	−0.47117 (18)	0.0458 (4)	
H8A	0.2989	0.1650	−0.5640	0.055*	
H8B	0.4376	0.1858	−0.4636	0.055*	
C9	0.41619 (18)	0.08813 (9)	−0.48056 (19)	0.0449 (4)	
C10	0.58017 (16)	0.10519 (8)	−0.23581 (17)	0.0376 (3)	
H10	0.6102	0.1460	−0.2707	0.045*	
C11	0.48458 (16)	0.12102 (8)	−0.13579 (17)	0.0373 (3)	
H11	0.4271	0.0836	−0.1336	0.045*	
C12	0.55952 (16)	0.13754 (8)	0.02285 (17)	0.0391 (4)	
C13	0.62934 (19)	0.19509 (9)	0.0542 (2)	0.0488 (4)	
H13	0.6259	0.2252	−0.0219	0.059*	
C14	0.7041 (2)	0.20809 (11)	0.1978 (2)	0.0631 (6)	
H14	0.7509	0.2468	0.2175	0.076*	
C15	0.7099 (2)	0.16440 (12)	0.3115 (2)	0.0664 (6)	
H15	0.7614	0.1732	0.4075	0.080*	
C16	0.6394 (2)	0.10777 (12)	0.2830 (2)	0.0618 (6)	
H16	0.6417	0.0784	0.3602	0.074*	
C17	0.56480 (19)	0.09428 (10)	0.1392 (2)	0.0498 (4)	
H17	0.5176	0.0556	0.1205	0.060*	
C18	0.70240 (17)	0.06669 (9)	−0.15516 (18)	0.0441 (4)	
H18A	0.6754	0.0232	−0.1361	0.053*	
H18B	0.7415	0.0868	−0.0591	0.053*	
N1	0.51362 (15)	0.06727 (7)	−0.36699 (16)	0.0446 (4)	
H1A	0.547 (2)	0.0287 (11)	−0.377 (2)	0.054*	
N2	0.40150 (15)	0.17695 (7)	−0.19801 (15)	0.0419 (3)	
H2A	0.358 (2)	0.1892 (10)	−0.130 (2)	0.050*	
O1	0.36881 (14)	0.05339 (7)	−0.58997 (15)	0.0643 (4)	

### Atomic displacement parameters (Å^2^)

**Table:**

	*U*^11^	*U*^22^	*U*^33^	*U*^12^	*U*^13^	*U*^23^
O2	0.050 (6)	0.142 (7)	0.066 (3)	0.000 (5)	0.023 (5)	0.029 (4)
O3	0.0556 (9)	0.074 (3)	0.064 (2)	0.031 (2)	0.0251 (14)	0.0246 (16)
C19	0.037 (3)	0.0839 (15)	0.023 (4)	0.004 (2)	0.0146 (19)	−0.003 (3)
C20	0.117 (3)	0.177 (7)	0.111 (5)	0.098 (4)	0.084 (4)	0.075 (4)
C21	0.136 (5)	0.134 (4)	0.149 (5)	0.038 (3)	0.098 (4)	0.013 (3)
O2'	0.050 (6)	0.142 (7)	0.066 (3)	0.000 (5)	0.023 (5)	0.029 (4)
O3'	0.0556 (9)	0.074 (3)	0.064 (2)	0.031 (2)	0.0251 (14)	0.0246 (16)
C19'	0.037 (3)	0.0839 (15)	0.023 (4)	0.004 (2)	0.0146 (19)	−0.003 (3)
C20'	0.117 (3)	0.177 (7)	0.111 (5)	0.098 (4)	0.084 (4)	0.075 (4)
C21'	0.136 (5)	0.134 (4)	0.149 (5)	0.038 (3)	0.098 (4)	0.013 (3)
C1	0.0471 (12)	0.0646 (14)	0.126 (2)	0.0075 (11)	−0.0103 (13)	−0.0144 (14)
C2	0.0544 (15)	0.093 (2)	0.151 (3)	0.0259 (14)	−0.0247 (17)	−0.018 (2)
C3	0.0797 (17)	0.0709 (16)	0.0892 (18)	0.0377 (14)	−0.0114 (14)	−0.0073 (13)
C4	0.0752 (14)	0.0485 (11)	0.0594 (12)	0.0135 (10)	0.0078 (10)	0.0012 (9)
C5	0.0473 (10)	0.0458 (9)	0.0454 (9)	0.0064 (8)	0.0065 (8)	−0.0004 (7)
C6	0.0406 (9)	0.0483 (9)	0.0396 (8)	0.0094 (7)	0.0032 (7)	−0.0037 (7)
C7	0.0396 (8)	0.0377 (8)	0.0422 (8)	0.0014 (7)	0.0047 (7)	−0.0014 (6)
C8	0.0521 (10)	0.0443 (9)	0.0354 (8)	0.0098 (8)	0.0002 (7)	−0.0033 (7)
C9	0.0450 (9)	0.0473 (9)	0.0390 (8)	0.0059 (8)	0.0038 (7)	−0.0091 (7)
C10	0.0399 (8)	0.0401 (8)	0.0303 (7)	0.0020 (7)	0.0040 (6)	−0.0026 (6)
C11	0.0390 (8)	0.0377 (8)	0.0335 (7)	0.0014 (6)	0.0059 (6)	0.0009 (6)
C12	0.0420 (9)	0.0437 (9)	0.0324 (7)	0.0070 (7)	0.0106 (6)	0.0003 (6)
C13	0.0576 (11)	0.0445 (9)	0.0417 (9)	0.0013 (8)	0.0073 (8)	−0.0023 (7)
C14	0.0690 (14)	0.0585 (12)	0.0541 (11)	0.0015 (10)	0.0008 (10)	−0.0151 (9)
C15	0.0723 (14)	0.0845 (16)	0.0354 (9)	0.0191 (12)	0.0005 (9)	−0.0094 (10)
C16	0.0681 (13)	0.0825 (15)	0.0356 (9)	0.0183 (12)	0.0142 (9)	0.0124 (9)
C17	0.0539 (11)	0.0547 (10)	0.0422 (9)	0.0037 (9)	0.0145 (8)	0.0081 (8)
C18	0.0403 (9)	0.0547 (10)	0.0340 (8)	0.0061 (8)	0.0033 (7)	−0.0011 (7)
N1	0.0464 (8)	0.0414 (8)	0.0398 (7)	0.0096 (6)	−0.0011 (6)	−0.0101 (6)
N2	0.0462 (8)	0.0440 (8)	0.0334 (7)	0.0106 (6)	0.0060 (6)	−0.0020 (6)
O1	0.0632 (9)	0.0623 (8)	0.0527 (8)	0.0199 (7)	−0.0136 (6)	−0.0251 (6)

### Geometric parameters (Å, º)

**Table:**

O2—C19	1.14 (3)	C6—C7	1.514 (2)
O3—C20	1.430 (8)	C7—N2	1.462 (2)
O3—C19	1.445 (15)	C7—C8	1.536 (2)
C19—C18	1.596 (7)	C7—H7	0.9800
C20—C21	1.298 (11)	C8—C9	1.505 (2)
C20—H20A	0.9700	C8—H8A	0.9700
C20—H20B	0.9700	C8—H8B	0.9700
C21—H21A	0.9600	C9—O1	1.234 (2)
C21—H21B	0.9600	C9—N1	1.332 (2)
C21—H21C	0.9600	C10—N1	1.460 (2)
O2'—C19'	1.30 (5)	C10—C18	1.524 (2)
O3'—C19'	1.20 (3)	C10—C11	1.545 (2)
O3'—C20'	1.474 (16)	C10—H10	0.9800
C19'—C18	1.294 (16)	C11—N2	1.470 (2)
C20'—C21'	1.130 (18)	C11—C12	1.512 (2)
C20'—H20C	0.9700	C11—H11	0.9800
C20'—H20D	0.9700	C12—C17	1.385 (2)
C21'—H21D	0.9600	C12—C13	1.385 (3)
C21'—H21E	0.9600	C13—C14	1.382 (3)
C21'—H21F	0.9600	C13—H13	0.9300
C1—C6	1.379 (3)	C14—C15	1.371 (3)
C1—C2	1.386 (4)	C14—H14	0.9300
C1—H1	0.9300	C15—C16	1.370 (3)
C2—C3	1.377 (4)	C15—H15	0.9300
C2—H2	0.9300	C16—C17	1.385 (3)
C3—C4	1.359 (3)	C16—H16	0.9300
C3—H3	0.9300	C17—H17	0.9300
C4—C5	1.385 (3)	C18—H18A	0.9700
C4—H4	0.9300	C18—H18B	0.9700
C5—C6	1.381 (3)	N1—H1A	0.88 (2)
C5—H5	0.9300	N2—H2A	0.89 (2)
			
C20—O3—C19	111.8 (4)	C7—C8—H8A	108.4
O2—C19—O3	132.9 (14)	C9—C8—H8B	108.4
O2—C19—C18	125.4 (17)	C7—C8—H8B	108.4
O3—C19—C18	101.1 (6)	H8A—C8—H8B	107.5
C21—C20—O3	117.8 (8)	O1—C9—N1	121.47 (16)
C21—C20—H20A	107.8	O1—C9—C8	120.70 (16)
O3—C20—H20A	107.8	N1—C9—C8	117.83 (15)
C21—C20—H20B	107.8	N1—C10—C18	106.70 (13)
O3—C20—H20B	107.8	N1—C10—C11	111.32 (13)
H20A—C20—H20B	107.2	C18—C10—C11	113.57 (13)
C19'—O3'—C20'	131.8 (12)	N1—C10—H10	108.4
O3'—C19'—C18	133 (2)	C18—C10—H10	108.4
O3'—C19'—O2'	102 (2)	C11—C10—H10	108.4
C18—C19'—O2'	124 (3)	N2—C11—C12	108.01 (13)
C21'—C20'—O3'	119.9 (12)	N2—C11—C10	109.60 (13)
C21'—C20'—H20C	107.4	C12—C11—C10	111.71 (13)
O3'—C20'—H20C	107.4	N2—C11—H11	109.2
C21'—C20'—H20D	107.4	C12—C11—H11	109.2
O3'—C20'—H20D	107.4	C10—C11—H11	109.2
H20C—C20'—H20D	106.9	C17—C12—C13	118.39 (16)
C20'—C21'—H21D	109.5	C17—C12—C11	120.79 (16)
C20'—C21'—H21E	109.5	C13—C12—C11	120.77 (15)
H21D—C21'—H21E	109.5	C14—C13—C12	120.41 (18)
C20'—C21'—H21F	109.5	C14—C13—H13	119.8
H21D—C21'—H21F	109.5	C12—C13—H13	119.8
H21E—C21'—H21F	109.5	C15—C14—C13	120.6 (2)
C6—C1—C2	120.1 (2)	C15—C14—H14	119.7
C6—C1—H1	120.0	C13—C14—H14	119.7
C2—C1—H1	120.0	C16—C15—C14	119.77 (19)
C3—C2—C1	120.5 (2)	C16—C15—H15	120.1
C3—C2—H2	119.8	C14—C15—H15	120.1
C1—C2—H2	119.8	C15—C16—C17	119.98 (19)
C4—C3—C2	119.9 (2)	C15—C16—H16	120.0
C4—C3—H3	120.1	C17—C16—H16	120.0
C2—C3—H3	120.1	C12—C17—C16	120.88 (19)
C3—C4—C5	119.8 (2)	C12—C17—H17	119.6
C3—C4—H4	120.1	C16—C17—H17	119.6
C5—C4—H4	120.1	C19'—C18—C10	116.5 (12)
C6—C5—C4	121.16 (18)	C10—C18—C19	112.1 (5)
C6—C5—H5	119.4	C19'—C18—H18A	107.9
C4—C5—H5	119.4	C10—C18—H18A	109.2
C1—C6—C5	118.56 (18)	C19—C18—H18A	109.2
C1—C6—C7	120.55 (18)	C19'—C18—H18B	105.9
C5—C6—C7	120.81 (15)	C10—C18—H18B	109.2
N2—C7—C6	109.00 (13)	C19—C18—H18B	109.2
N2—C7—C8	111.87 (14)	H18A—C18—H18B	107.9
C6—C7—C8	107.62 (14)	C9—N1—C10	125.81 (15)
N2—C7—H7	109.4	C9—N1—H1A	116.7 (13)
C6—C7—H7	109.4	C10—N1—H1A	117.1 (13)
C8—C7—H7	109.4	C7—N2—C11	117.82 (13)
C9—C8—C7	115.33 (15)	C7—N2—H2A	106.8 (13)
C9—C8—H8A	108.4	C11—N2—H2A	107.3 (13)
			
C20—O3—C19—O2	−7 (3)	C10—C11—C12—C13	70.74 (19)
C20—O3—C19—C18	−178.8 (7)	C17—C12—C13—C14	1.1 (3)
C19—O3—C20—C21	−97.5 (9)	C11—C12—C13—C14	−176.07 (17)
C20'—O3'—C19'—C18	177 (2)	C12—C13—C14—C15	−0.3 (3)
C20'—O3'—C19'—O2'	3 (4)	C13—C14—C15—C16	−0.9 (3)
C19'—O3'—C20'—C21'	149 (2)	C14—C15—C16—C17	1.2 (3)
C6—C1—C2—C3	−0.7 (5)	C13—C12—C17—C16	−0.8 (3)
C1—C2—C3—C4	−0.7 (5)	C11—C12—C17—C16	176.38 (16)
C2—C3—C4—C5	1.3 (4)	C15—C16—C17—C12	−0.3 (3)
C3—C4—C5—C6	−0.5 (3)	O3'—C19'—C18—C10	−167 (2)
C2—C1—C6—C5	1.5 (4)	O2'—C19'—C18—C10	5 (4)
C2—C1—C6—C7	−175.1 (3)	O3'—C19'—C18—C19	−151 (26)
C4—C5—C6—C1	−0.9 (3)	O2'—C19'—C18—C19	21 (22)
C4—C5—C6—C7	175.72 (17)	N1—C10—C18—C19'	69.2 (14)
C1—C6—C7—N2	−139.3 (2)	C11—C10—C18—C19'	−167.8 (14)
C5—C6—C7—N2	44.1 (2)	N1—C10—C18—C19	67.8 (6)
C1—C6—C7—C8	99.2 (2)	C11—C10—C18—C19	−169.1 (6)
C5—C6—C7—C8	−77.4 (2)	O2—C19—C18—C19'	−140 (25)
N2—C7—C8—C9	77.82 (19)	O3—C19—C18—C19'	32 (23)
C6—C7—C8—C9	−162.47 (14)	O2—C19—C18—C10	24.1 (19)
C7—C8—C9—O1	117.5 (2)	O3—C19—C18—C10	−163.3 (5)
C7—C8—C9—N1	−62.1 (2)	O1—C9—N1—C10	177.30 (18)
N1—C10—C11—N2	−81.30 (16)	C8—C9—N1—C10	−3.1 (3)
C18—C10—C11—N2	158.24 (14)	C18—C10—N1—C9	−168.04 (17)
N1—C10—C11—C12	159.02 (13)	C11—C10—N1—C9	67.5 (2)
C18—C10—C11—C12	38.57 (19)	C6—C7—N2—C11	174.23 (14)
N2—C11—C12—C17	132.98 (17)	C8—C7—N2—C11	−66.87 (19)
C10—C11—C12—C17	−106.41 (18)	C12—C11—N2—C7	−168.61 (14)
N2—C11—C12—C13	−49.9 (2)	C10—C11—N2—C7	69.48 (18)

### Hydrogen-bond geometry (Å, º)

**Table:**

*D*—H···*A*	*D*—H	H···*A*	*D*···*A*	*D*—H···*A*
N1—H1*A*···O1^i^	0.88 (2)	1.97 (2)	2.846 (2)	174 (1)

Symmetry code: (i) −*x*+1, −*y*, −*z*−1.
